# Human DNA from the oldest Eneolithic cemetery in Nalchik points the spread of farming from the Caucasus to the Eastern European steppes

**DOI:** 10.1016/j.isci.2024.110963

**Published:** 2024-10-16

**Authors:** K.V. Zhur, F.S. Sharko, M.V. Leonova, A. Mey, E.B. Prokhortchouk, V.A. Trifonov

**Affiliations:** 1 Federal Research Centre «Fundamentals of Biotechnology» of the Russian Academy of Sciences, Moscow, Russian Federation; 2 A.MEY Eventmanagement GmbH & Co. KG, Salzatal, Germany; 3 Institute for the History of Material Culture, Russian Academy of Sciences, St Petersburg, Russian Federation

**Keywords:** Human geography, Genomics, Anthropology, Archeology

## Abstract

The Darkveti-Meshoko culture (c.5000–3500/3300 BCE) is the earliest known farming community in the Northern Caucasus, but its contribution to the genetic profile of the neighboring steppe herders has remained unclear. We present analysis of human DNA from the Nalchik cemetery—the oldest Eneolithic site in the Northern Caucasus—which shows a link with the LowerVolga’s first herders of the Khvalynsk culture. The Nalchik male genotype combines the genes of the Caucasus hunter-gatherers, the Eastern hunter-gatherers and the Pre-Pottery Neolithic (PPN) farmers of western Asia. Improved comparative analysis suggests that the genetic profile of certain Khvalynsk individuals shares the genetic ancestry of the Unakozovo-Nalchik type population of the Northern Caucasus’ Eneolithic. Therefore, it seems that in the first half of the 5th millennium BCE, cultural and mating networks helped agriculture and pastoralism spread from West Asia across the Caucasian, into the steppes between the Don and the Volga in Eastern Europe.

## Highlights

• An aDNA from the Caucasus links the Volga’s first herders to farmers of West Asia
• An ancient genome points the spread of farming through the Caucasus to the Steppe

## Graphical abstract

**Figure:**
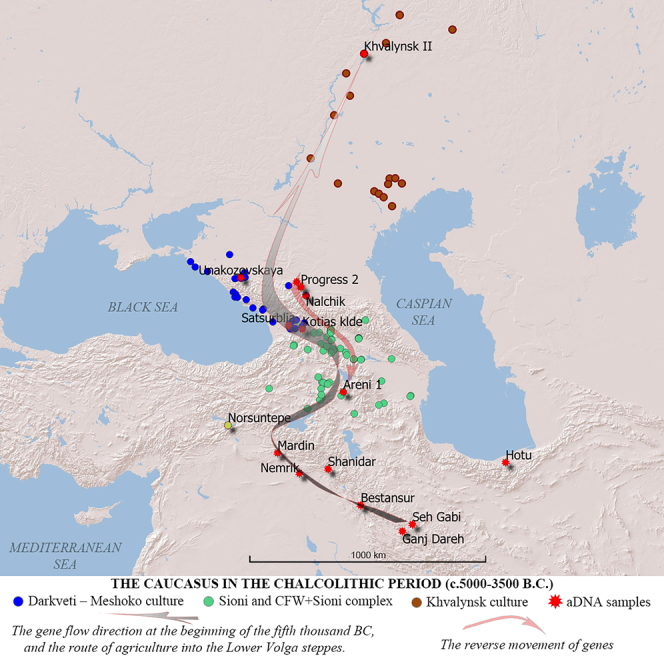

## Introduction

At the end of the seventh-early sixth millennia BC the West Asian Neolithization wave reached the plains of the Southern Caucasus where Neolithic sites of the Shulaveri-Shomutepe-Aratashen type spread across the Kura and Araxes valleys.^1^^,^^2^^,^^3^^,^^4^^,^^5^ Because of a lack of radiocarbon dates, it is difficult to argue that Neolithic sites appeared in Western Georgia at the same time as well, but it cannot be ruled out.^6^ However, there are no archaeological indications that the Southern Caucasus cultures of the Neolithic type spread to the Northern Caucasus. All this indicates that the Main Caucasus Range remained impervious to these cultures. The paradox of the situation is that since the Mesolithic^7^^,^^8^ and in fact until the end of the sixth millennium BC, population density in the North Caucasus was apparently extremely low. No sites dating from the seventh to sixth millennia BC are known in this region. In this context, the appearance of a large number of Eneolithic sites such as Darkveti and Meshoko in the Northwest Caucasus at the beginning of the 5th millennium BC looked like a demographic explosion. It was the settlement of this territory by people who were the first to bring here the main transformational technologies of the Neolithic way of life—farming and cattle breeding.

The question of the origin of this population is equally relevant both for reconstructing the process of Neolithization in the North Caucasus and for understanding the subsequent spread of elements associated with this type of economy in the East European steppe areas between the Don and the Volga adjacent to the Caucasus.

This issue is difficult to resolve because of the small number of anthropological remains associated with sites of the Darkveti-Meshoko type that could be available for genetic analysis. There are about three dozen such sites, and there are only three known burials, and only in one burial (Unakozovo cave) were the remains of people in the first degree of kinship subjected to genetic analysis.^9^

The goal of this study is to present for the first time the results of genomic analysis of ancient human DNA from the Nalchik burial ground, which is the earliest (c. 5000/4800 cal. BC) collective (121 burials) Eneolithic burial ground in the North Caucasus.

Excavated in 1929, the cemetery was located on the border between the foothills of the central North Caucasus and the Fore-Caucasian edge of the Eurasian steppe. The peculiarity of the monument lies in its cultural features, which make it possible to attribute it to the Caucasian group of the Darkveti-Meshokov tradition^10^ and in its connection with sites of the Khvalynsk type found in the Volga Basin ^11^^,^^12^. During this period, populations in the North Caucasus were just beginning to master farming^13^ and animal husbandry,^14^ while populations in the neighboring steppes of the Lower Volga and Lower Don steppes were just beginning to raise cattle and sheep/goats.^15^ All this occurred about 1,000 years before the Maikop culture in the Caucasus (3700–3000/2900 BCE) and almost 1,500 years before the Yamnaya culture in the steppes. In the subsequent Bronze Age, the Yamnaya people determined the trends of cultural and economic development, as well as the Eurasian genetic landscape.^16^

The role assigned to the Eneolithic Darkveti-Meshoko population in this cultural and historical process was key rather than supporting, because without this population’s contribution it is not possible to correctly assess the main historical trends in the economic and genetic history of the Caucasus and the adjacent steppe in the Eneolithic and then in the Early Bronze Age.

We think that the Darkveti-Meshoko population not only brought a Neolithic lifestyle to the Northern Caucasus but also transferred skills of producing economy to the Volga-Don steppe population.

In the context of this hypothesis verification, we present the results of the genome-wide analysis of the individual from grave 42 at the Nalchik cemetery.

## Results and discussion

### Genome-wide sequencing and analysis of the uniparentally inherited markers

As a result of genome-wide sequencing of the library of NL1.2.2 aDNA fragments, more than 155 million reads were generated and 452,778 single nucleotide polymorphisms (SNPs) were identified (Tables S3 and S4). The data suggested that the genome belonged to a man, his mitochondrial haplogroup was T2c1a1, and the Y chromosome haplogroup was R1b1 (R-L1068/etc∗ (xL502,CTS10490, FGC20996). No contamination of the sample was detected according to such parameters as the degree of heterozygosity of mtDNA and X chromosome (Tables S5 and S6).

### Genetic clustering of man from a burial of the Eneolithic period in Nalchik

**Figure 1:**
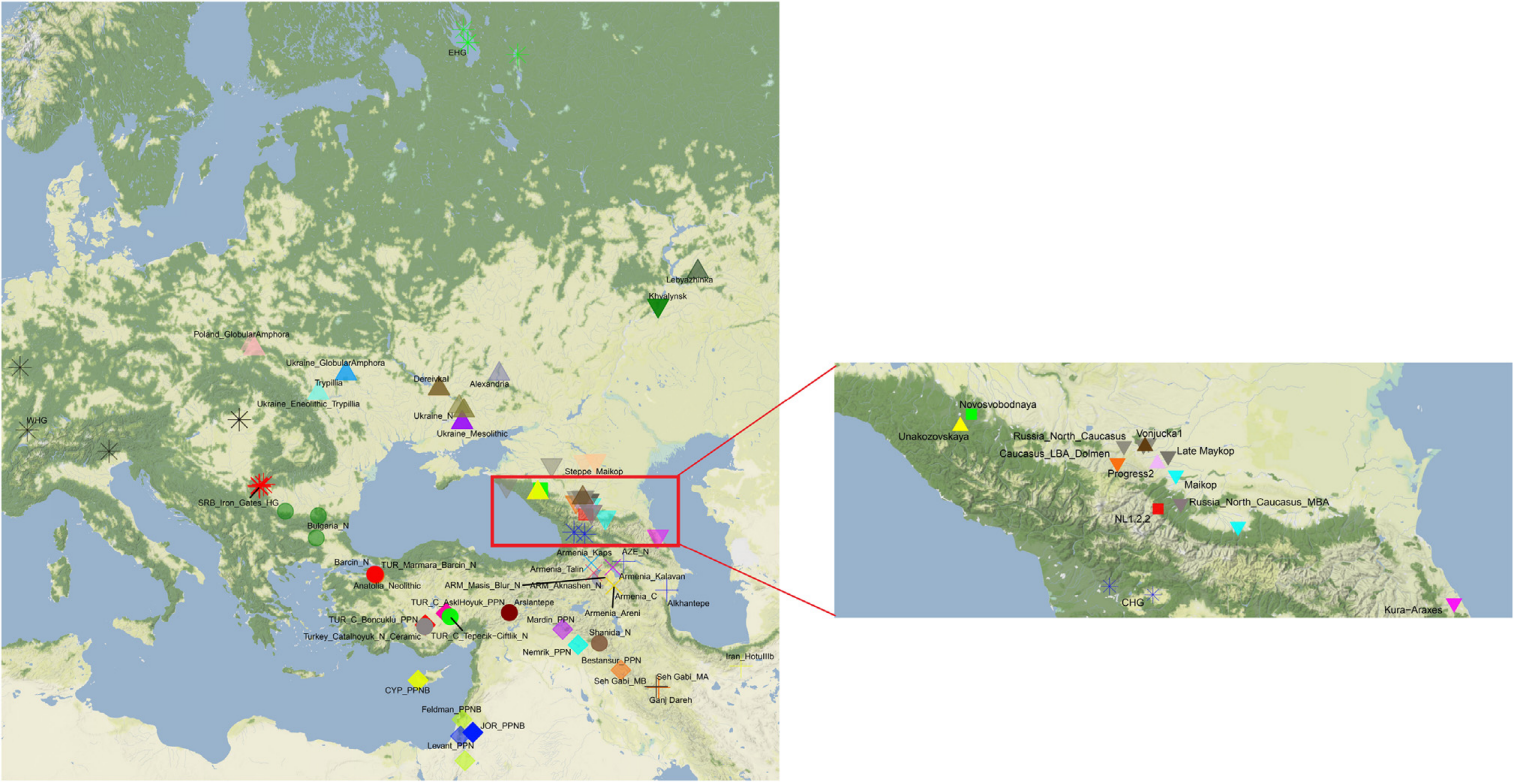
Map of samples and sites mentioned in this study The zoomed map shows the location of individuals from various sites in the Caucasus.

To assess the genetic affinities of the ancient individual qualitatively we first performed principal-component analysis (PCA)^17^ and ADMIXTURE analysis.^18^ A list of samples is presented in Table S2, and their geographical location presented in Figure 1.

**Figure 2:**
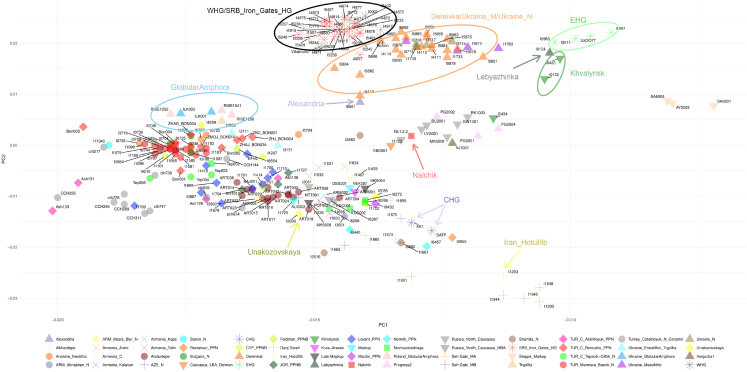
PCA results The genome of the Nalchik man was projected onto the ancient populations.

The PCA space was constructed using modern samples, and Nalchik man was included along with a collection of ancient genomes, which were then projected onto the first two eigenvectors. (Figure 2). The location of ancient genomes on PCA map correlates with the geographical coordinates of the corresponding archaeological sites. The PC1 axis mainly coincides with the West-East direction and the PC2 with the North-South. Based on PCA plots, we observe that the genome of Nalchik man (sample coordinates PC1: −0.0131, PC2: 0.0018) takes on intermediate positions between ancient individuals from the West Eurasian steppe and ancient individuals from Caucasus region. Samples from North Caucasus piedmont steppe, Eneolithic individuals from the sites of Progress 2 (the most ancient sample with dating of 4994-4802 calBCE) and Vonyuchka 1 (4337-4177 calBCE), along with one steppe individual from Khvalynsk II (ID I0434, 5198-4853 calBCE)^19^ are positioned close to Nalcnik man in PC1-PC2 space. Unakozovskaya cave samples although belonging to the same Darkveti-Meshoko culture resides in a cluster of Caucasus region.

**Figure 3:**
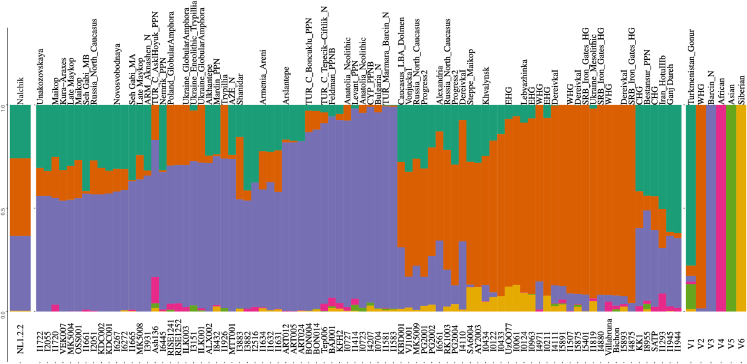
Results of the Admixture analysis For representatives of ancient populations whose genomes were used to assess the genetic origin of Nalchik man (k = 6). Samples I10409, I0001, I11949, HGDP00920.HO, HGDP00811.HO, and HGDP00832.HO constitute V1, V2, V3, V4, V5, and V6 respectively.

Figure 3 presents the results of an Admixture analysis with the number of ancestral populations equal to six (*K* = 6). The genome of the Nalchik Man and individuals from Unakozovskaya cave were analyzed in the context of the Caucasus hunter-gatherers (CHG) cluster (Kotias, Satsurbia etc), ancient samples from the South Caucasus (Alkhantepe, Arslantepe, Armenia_Chalcolithic), individuals of the West Eurasian steppe (Khvalynsk, DereivkaI, Lebyazhinka VI, Ukraine_Mesolithic), and other samples from adjacent areas of previous, synchronous, and later periods of time.

Admixture analysis has shown that the ancient individuals from Unakozovskaya cave have mixed ancestry mostly derived from sources related to the Anatolian Neolithic (purple), CHG/Iran Neolithic (dark green), and EHG\WHG (orange) in the Admixture plot. This is consistent with data previously published by Wang et al.,^9^ who suggested the presence of this mixed ancestry north of the Caucasus as early as ∼6,500 years ago. Similar ancestry profiles were reported for Anatolian and Armenian Chalcolithic (Areni-1 cave) ancient samples.^9^

Сompared to individuals from Unakozovskaya cave the Nalchik man shows the enrichment of the V2 (orange) component, which is dominant in the West Eurasian steppe, like EHG, WHG, SRB_Iron_Gates_HG, Khvalynsk II, DereivkaI, Lebyazhinka IV, and other samples. The increased presence of the steppe component in the genome of the Nalchik Man is expected, as there would have been greater interaction with the steppe in the border region (Nalchik cemetery) compared to the mountain zone (Unakozovskaya cave).

To test that the Nalchik man has the excess of alleles shared with EHG/WHG compare to individuals from the Unakozovskaya cave, we performed F4-statistics in the form of (Yoruba, West Eurasian steppes; Unakozovskaya, Nalchik). The statistics produced significantly positive *Z* scores for EHG and WHG (Table S7). Similar results were obtained when we tested the excess of alleles shared with EHG/WHG between Nalchik man and individuals from Areni-1 cave (Armenian Chalcolithic) by F4-statistics of the form of (Yoruba, West Eurasian steppes; Armenia_Areni, Nalchik).

#### Outgroup F3 statistics

**Figure 4:**
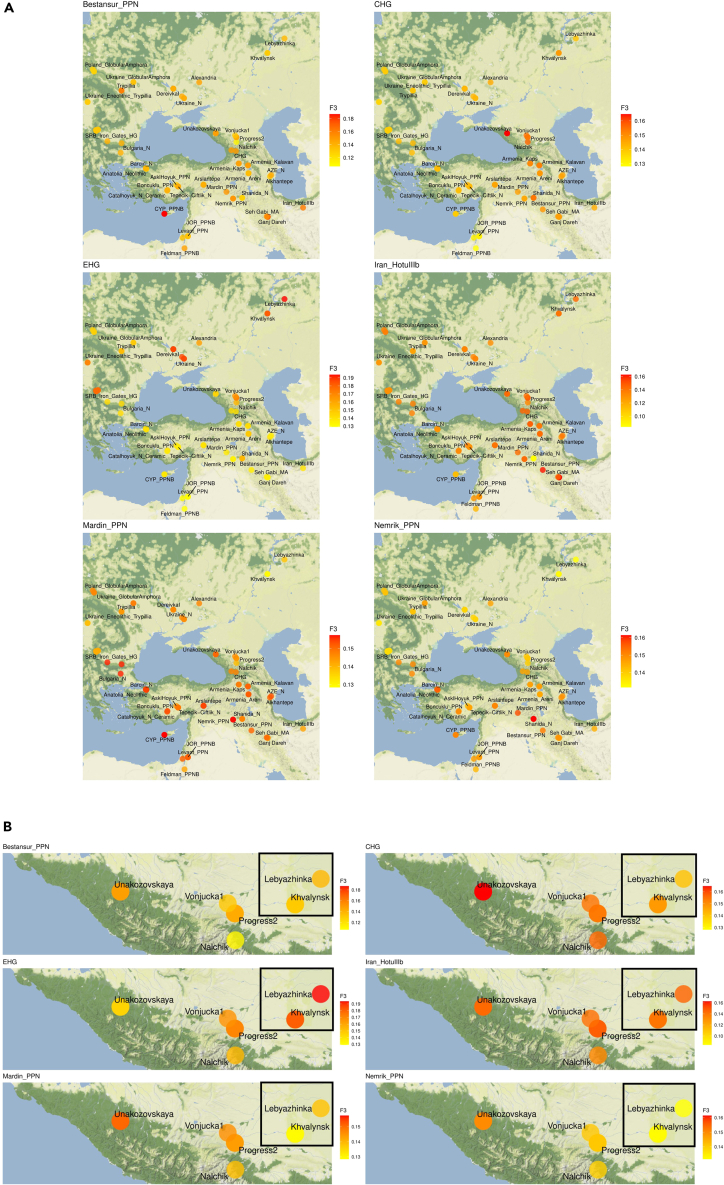
The geographical distribution of outgroup F3 statistics to measure the genetic closeness of populations to Darkweti-Meshoko and other cultures (test) (A) Outgroup F3 statistics (test, candidates; Yoruba) were obtained using Candidate genomes from steppe (EHG), Caucasus (CHG and Iran_HotuIIIb), and Neolithic populations. Higher F3 statistics (red colors) indicate more shared drift with the respective group in Candidates. All F3 statistics can be found in Table S8. (B) The zoomed map shows the location of individuals from Lebyazhinka and Khvalynsk.

We used outgroup F3 statistics to find the most relevant populations that might be used in further qpAdm modeling of Nalchik man genome. According to Admixture analysis, apparent candidates were individuals from steppe (EHG), from Caucasus region (CHG and Iran_HotuIIIb), and Neolithic people (candidates). To measure genetic affinity of the ancient populations to Darkveti-Meshoko and other cultures (test) that are in a scope of our study, we performed the statistic F3(test, candidates; Yoruba). The results (Figure 4) confirm the assumption that particular Pre-Pottery Neolithic (PPN) populations interacted with an ancestors of Nalchik man (more candidates/test combinations are given in Figure S5; Table S8). Of special note is an observation that steppe populations like Khvalynsk and Lebyazhinka produce significant F3 values with PPN’s. Moreover Lebyazhinka, which is dated 500 years before Khvalynsk and located 300 km northeast of it, shows stronger affinity to Mardin_PPN than Khvalynsk. Presumably, the impulse of PPN’s reached these steppe regions but in the time course was diluted by local archaic EHG’s.

Thus, ancient human DNA from the Nalchik cemetery, the oldest Eneolithic cemetery in the North Caucasus, and the Lebyazhinka site suggest that the West Asian PPN genetic heritage reached Eurasian steppe at least by the early 5th millennium BCE.

#### Modeling of the Nalchik Man genome, individuals from the Unakozovskaya cave, and Armenian Chalcolithic representatives

**Figure 5:**
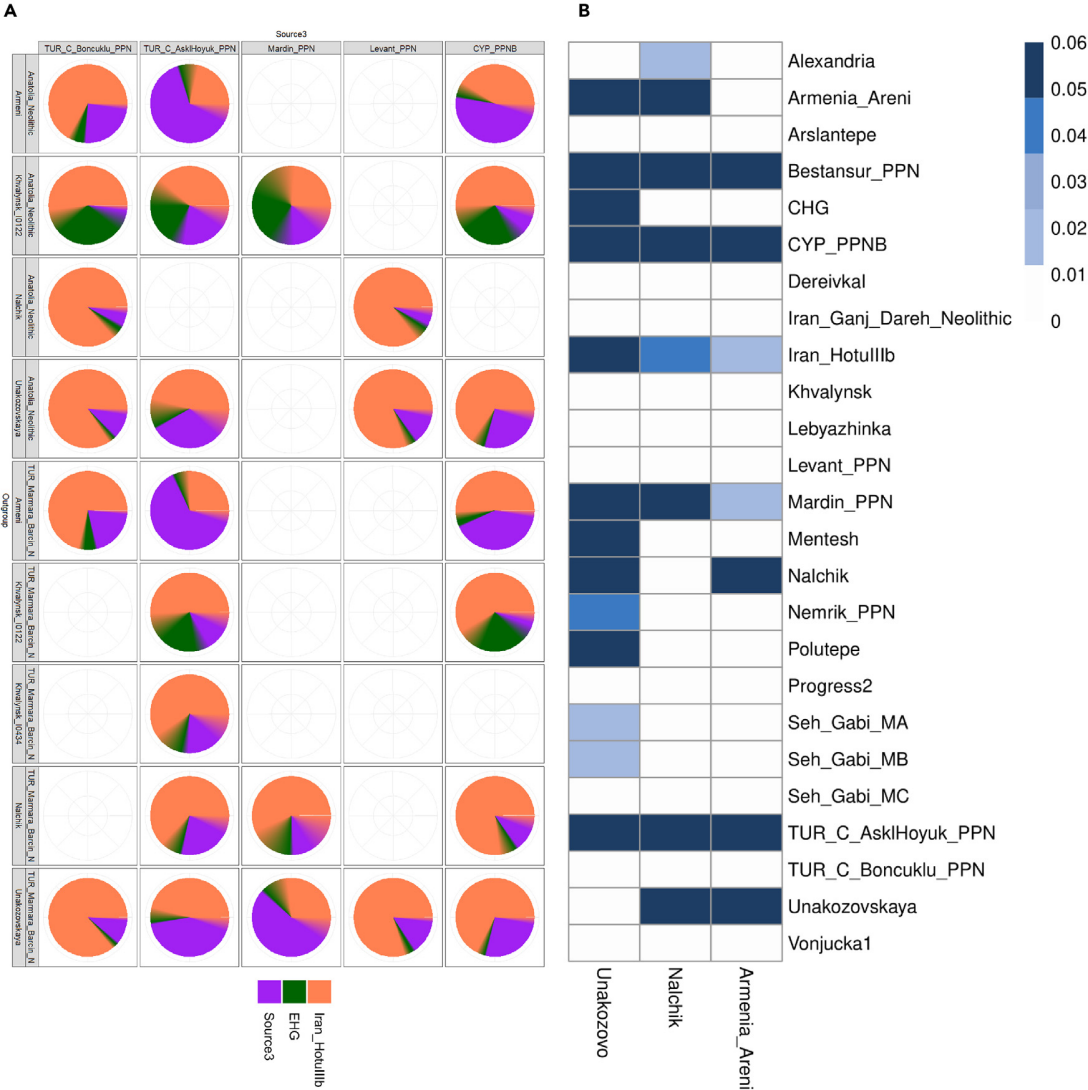
Modeling the ancestry of the Nalchik man (A) Individuals from the Unakozovskaya cave and Armenian Chalcolithic representatives from the genomes of ancestral populations. Colored squares depict whether a particular target group can be modeled using a single source group. (B) Individuals from the Unakozovskaya cave, Armenian Chalcolithic representatives, and individuals from the Khvalynsk burial site as a three-way admixture. Empty cells suggest that no significant statistics were detected for this combination of the source and the outgroup. Standard errors of statistics are given as a blurring border between sectors with distinct colors.

The proportions of ancestry from chronologically proximal and distal populations that contributed to the Nalchik Man genome were determined by qpAdm. This model enables testing for continuity between CHG/PPN and other groups from surrounding areas during preceding, synchronous, and later periods of time. The results of a simple one-way modeling showed that Nalchik Man ancestry, individuals from the Unakozovskaya cave, and from Armenian Chalcolithic culture can be modeled with Bestansur_PPN, CYP_PPNB, and TUR_C_AsklHoyuk_PPN as a single source (Figure 5A; Table S9). Mardin_PPN also provides a fit but only for Nalchik man and individuals from the Unakozovskaya cave. However, we found that only individuals from the Unakozovskaya cave showed high genetic affinities with more ancient Caucasus representatives such as CHG, Iran_HotuIIIb, Mentesh, and Polutepe.

To provide better fit to the model the second source population was included into the analysis. The statistics of the 2-source modeling, where the ancient Caucasus source was represented by CHG or Iran-Hottulllb, support that the Nalchik Man genome has a significant input from the steppe (up to 25.2%), particularly from EHG (see Table S8). It is worth mentioning that the ancestry of Chalcolithic individuals from Armenia also corresponds to the hypothetical genetic connections with Unakozovskaya cave individuals (up to 83.3%) and people with steppe ancestry (up to 23.5%) (see Table S10).

Archeology inevitably proved the fact that Darkveti-Meshoko culture mastered farming and animal husbandry. To detect genetic sources of the Neolithization, genomes of prepottery farmers were used in qpAdm modeling along with CHG and EHG. The results of a three-way admixture with Iran_HotuIIIb as the first source, EHG ancestry as the second and different PPN ancestry as the third source with various combinations of Anatolian Neolithic populations as outgroup are presented in Figure 5b and Table S12(11), Table S13(12). The maximum proportions of pre-pottery Neolithic ancestry that contributed to the Nalchik man genome, individuals from the Unakozovskaya cave, and Armenian Chalcolithic representatives were established for CYP_PPNB, TUR_C_Boncuklu_PPN, Mardin_PPN, and TUR_C_AsklHoyuk_PPN when TUR_Marmara_Barcin_N or Anatolia_Neolithic were used as outgroup populations (Figure 5B).

Swaping PPN and Anatolia Neolithic as “source” and “outgroup” resulted in a decrease of statistically significant combinations, but for those that did produce *p* values > 0.05, the input of Anatolia Neolith consisted just 2–12% when PPN was used as a background.

Modeling of Nalchik man and Unakozovo cave sample using four (CHG, EHG, PPN, and Anatolian Neolith) or five sources (CHG, EHG, PPN, Anatolian Neolithic, and SRB_HG) did not produce statistically significant results (data not shown).

#### Modeling the ancestry of individuals from Khvalynsk II

Taking into account the archaeologically recorded connection of Darkveti-Meshokov tradition^10^ with sites of the Khvalynsk type,^12^ we assessed the possibility of contacts between Dаrkveti-Meshoko\Nalchik man with representatives of the West Eurasian steppe cultures at genetic level. Admixture F3-statistics of the form F3(X, Y; target) with the Khvalynsk II (Samara Oblast, Volga River Valley) as target resulted in significantly negative *Z* scores when the Nalchik Man (|Z| = 3.38) or Unakozovskaya cave samples (|Z| = 4.22) were used as the first source and hunter gatherers from West Eurasian steppes, like Russia_HG_Samara (Lebyazhinka IV) as the second source (Table S13) suggesting both Unakozovo cave and Nalchik men along with steppe people could be a proxy for shaping genetic portraits of Khvalynsk population.

The genetic interaction between individuals of the Darkveti-Meshoko Eneolithic culture and people from the steppe could have resulted in the transfer of a farmer-related genetic component to their descendants. To test this hypothesis, 3-source qpAdm analysis of Khvalynsk individuals^19^ was performed. Two sources, EHG and Iran_HotuIIIb, were fixed and the third source was assessed among all possible farmer-related individuals including PPN genomes. PCA analysis showed heterogeneity of Khvalynsk samples: I0434 was very close to the North Caucasus cluster that contains Eneolithic individuals from the sites of Progress 2 (2476-2303 calBCE (MAMS-29815)) and Vonyuchka 1 (4337-4177 calBCE (MAMS-29823)), I0433 was close to steppe and EHG samples while I0122 took an intermediate position between I0433 and I0434. Three source qpAdm confirmed that these three samples although being attributed to the same archeological culture, have very different genetic ancestry background. I0433 could not be modeled by three source qpAdm. I0434 and I0122 show a gradient of EHG ancestry—8% and 28%, respectively. Maximum PPN ancestry was detected in I0434 sample- 68%, and only 25% in I0122 (Figure 5B; Table S11).

According to archaeological context I0122 (4936-4730 calBCE) probably is a high-status individual, that could be regarded as a founder of an elite “yellow” group of patrilineally related families. His Y chromosome haplotype—R1b1, his MtDNA haplotype—H2a1, is unique in the Samara series. Individuals I0433 (mtDNA U5a1, Y-chrom R1a- M459) and I0434 (mtDNA U4d, Y-chrom Q1- L472) was accompanied by a very modest set of grave goods without sacrificial animals; individual I0433 had no grave goods at all. Both belonged to the “gray” family group.^15^

Based on results of PCA, Admixture, and qpAdm modeling, it is clear that Khvalynsk culture individuals were genetically heterogenous with a following components gradient: increasing of EHG and decreasing of PPN (CYP_PPNB, Mardin_PPN, and TUR_C_AsklHoyuk_PPN) in I0434-I0122-I0433 series of samples. Ancient CHG (Kotias/Satsurbia) or similar IranHottulllb components were detected in all three samples.

#### IBD analysis of Khvalynsk, Unakozovo, and Nakchik samples

**Figure 6:**
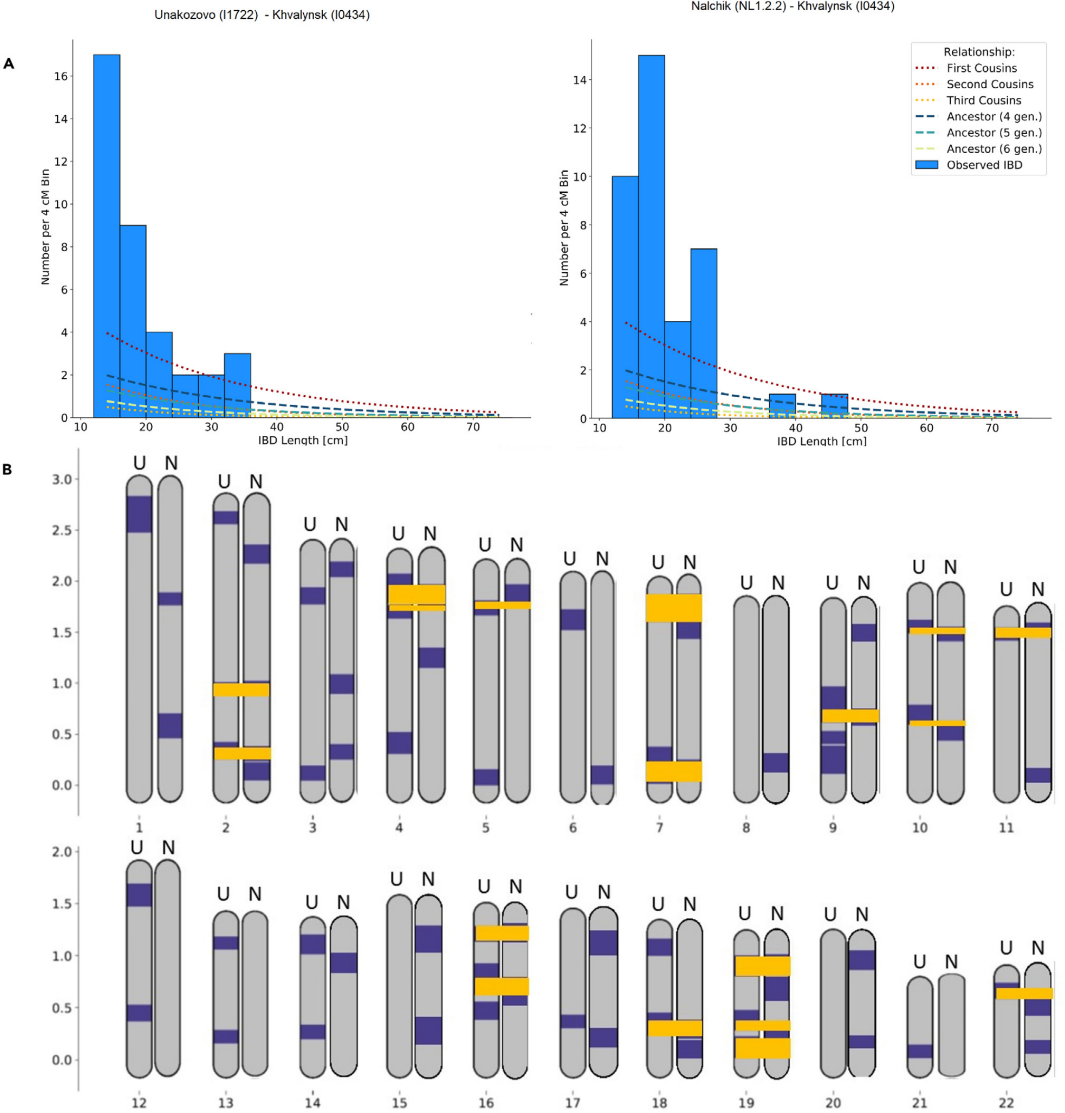
IBD inferred (A) Histogram of inferred IBD segment lengths, as well as theoretical expectations for various types of relatives. (B) Plot of all inferred IBD segments longer than 12 cM. Common haploblocks between Unakazovo and Khvalynsk 434 (U), Nalchik and Khvalynsk 434 (N) are marked in yellow.

To test recent gene flow from the Darkveti-Meshoko culture to Khvalynsk, we analyzed the imputed genomes of Khvalynsk_I0434, Unakozovo cave, and Nalchik man (see Figure 6A). Our findings indicate that the Nalchik Man and the Khvalynsk_I0434 sample share two recent haploblocks (44.32 and 36.29 cM, on chromosomes 7 and 19, respectively). The overall distribution of common haplotypes between the samples suggests that there are at least 5 edges on a pedigree graph between these two samples. There are some common haplotypes between the Unakozovo cave and Khvalynsk_I0434 sample, but there is no evidence of recent mating events (maximum identity by descent [IBD] 35.94 cМ). When comparing the Unakozovo cave and Nalchik man, a very short IBD (18.76 cM) are observed. The haplotypes of these genomes have very little overlap, as shown in Figure 6B. The data suggest that the Khvalynsk_I0434 genome had independent input from the ancestors of Nalchik man and Unakozovo cave individual, with more recent gene flow from Nalchik man. No signs of recent interaction with Unakozovo or Nachik man were detected by IBD analysis for two other Khvalynsk samples I0122 or I0433 (Table S14).

### Conclusion

The DNA sequence analysis of the Nalchik man, using a range of methods including PCA, Admixture, F3 and F4 statistics, qpAdm, and IBD tests with imputed genomes, provides statistical evidence for the occurrence of the first wave of steppe Neolithization during the period of 5000-4500 BCE. The conventional model predicted that Nalchik ancestry could be modeled with three sources—CHG, EHG, and Neolithic. However, we found that PPN populations, in contrast to Anatolian Neolithic populations, had the most significant impact on the genome. We specifically focused on the presence of the PPN component and also analyzed other known genomes of Caucasus and steppe culture representatives that may be closely related to the Darkveti-Meshoko culture.

**Figure 7:**
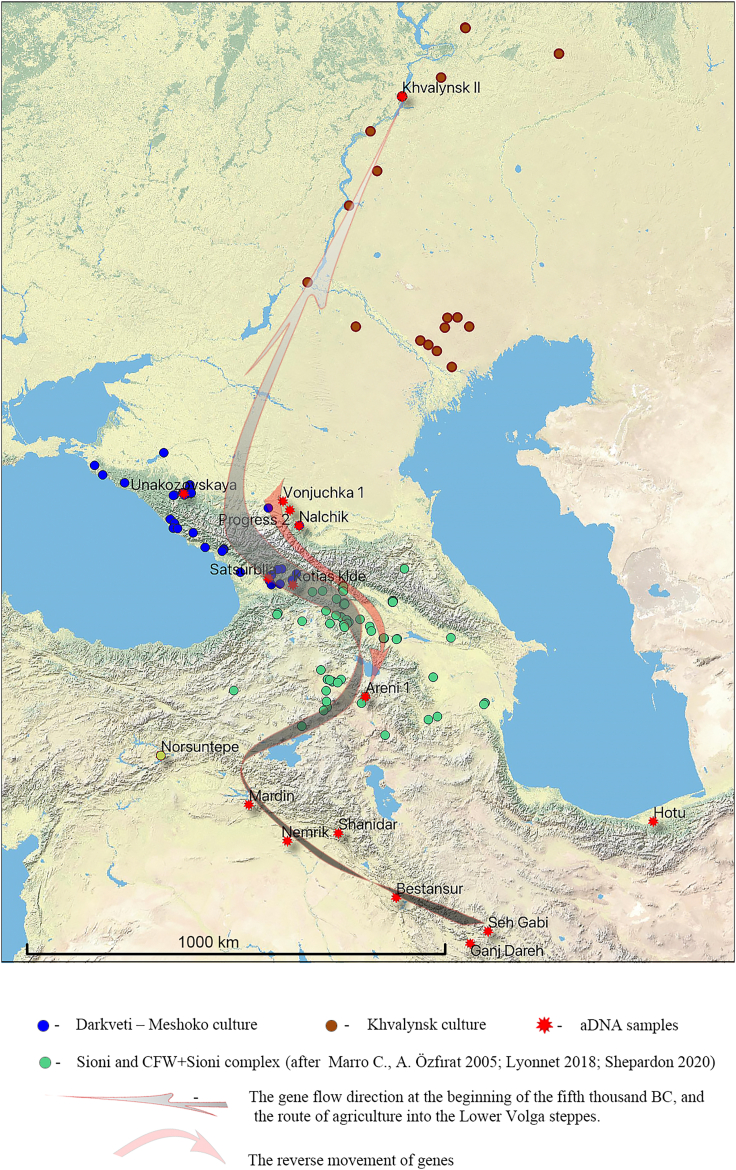
The Caucasus in the Chalcolithic period (c. 5000-3500 B.C.)

The results of the paleogenetic analysis of the human sample from the Eneolithic cemetery in Nalchik offer a new perspective on the population that had already achieved a Neolithic way of life and the stream of this population to the northern slopes of the Caucasus and then northward to the Caucasus piedmont steppe and the Lower Volga basin which occurred in the first half of the fifth millennium BCE (Figure 7).

Contrary to expectations, the Nalchik individual genetically closer to earlier population of Northern Mesopotamia and Zagros (eighth–seventh millennia BCE) which lived far from the Caucasus (PPN/N) than to the ancestry composition of the neighboring Neolithic population of the Southern Caucuses in the sixth millennium BCE (sites of the Shulavery-Shomutepe-Aratashen type).

The cultural and historical context of this genetic landscape is not fully understood yet, and the trajectory of gene flow from Northern Mesopotamia to the Northern Caucasus and then further to the Eastern European steppe can now be shown only as a dotted line. Substantial gaps in the archeological material represent a key issue in reconstructing the trajectory of this movement in the period between the eighth millennium BC and the fifth millennium BCE.

Archaeological links between the Northern Caucasus Eneolithic population and PPN societies of Northern Mesopotamia are illusory. The only thing that these populations share are traditions of making stone bracelets^20^ that presently can hardly be regarded as interrelated, considering a substantial chronological gap.

**Figure 8:**
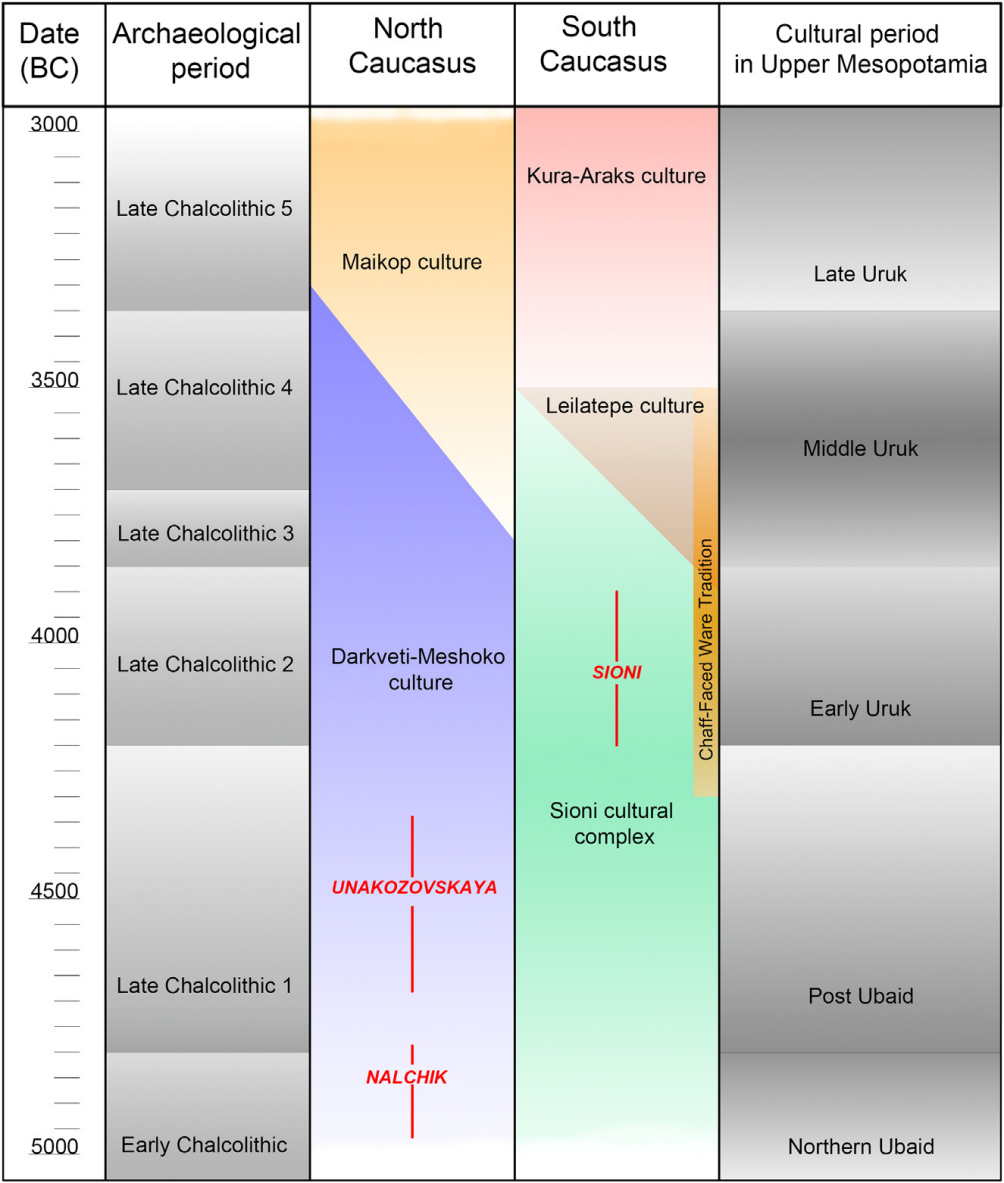
Chronology of the 5^th^ to the 4^th^ millennia BC in the Caucasus

Real cultural links among the Northern Caucasus, the Southern Caucasus, and the Northern Mesopotamia can be traced only starting from the period between the Ubaid and the Uruk according to the Mesopotamian chronology (post-Ubaid period), which, in the regions north of the Upper Mesopotamia, corresponds to the late Chalcolithic (с. 4500–3800/3500 BCE)^21^ (Figure 8).

At that time distinctive comb-stamped ware^22^ known at the late Chalcolithic (LC1) settlements in the Araxes valley (Ovçular Tepesi) and in the Upper Euphrates (Norşuntepe)^23^^,^^24^ appeared at the Eneolithic settlements in the Northern Caucasus (Meshoko and Myskhako). In addition, the settlements dating to the first half of the fifth millennium BC located in the Southern and Northern Caucasus are linked by pottery of the Sioni type (Zamok, Vorontsovskaya peschera, and Mentesh Tepe).^25^^,^^26^ The Areni cave site in Armenia is also ascribed to this late Chalcolithic tradition,^27^^,^^28^ this fact explains genetic similarity between the buried individuals in Areni and those from the Nalchik cemetery. More importantly, the presence of the component of steppe ancestry (EHG) in the genetic profile of the Areni individuals can be used as an indicator of two-gene flow canal with a reverse movement of genes from the Southern Caucasus to the Northern Caucasus and then back again.

Given the dating of the Nalchik cemetery, the emergence of the Trans-Caucasus route for geographical distribution of the population that had producing economy skills must precede the sites of the Areni type and must be dated not later than the early fifth millennium BC. However, the archaeological context of the end of the sixth–first half of the fifth millennia BCE in the Southern Caucasus, in particular, the mountain areas, remains largely unexplored. It is fair to assume that after the Mesolithic pause the northward movement of the post-Neolithic groups was basically reoccupation adapted to various landscapes.^29^^,^^30^

One of the results of this process is belated Neolithization of the Northern Caucasus in the first half of the fifth millennium BCE which was followed by Neolithization of the adjacent steppe as far as the Lower Volga in the Eneolithic.

Genetic heterogeneity of the Khvalynsk individuals who represent various combinations of basic steppe genetic ancestry (EHG) in combination with Caucasus (CHG) and West Asian farmer-related components demonstrates relatively low intensity of the mating links between the Northern Caucasus and the steppe communities. Still these contacts were sufficient to transfer herding skills and lead to irreversible changes in the lifestyle in the steppe.

Genetic infiltration of the Caucasus population into the steppe in the first half of the fifth millennium BC was, most likely, not the first and, more importantly, was not the only one. Judging by the combination of the EHG and the CHG components in some Khvalynsk individuals, the latter component could reach the steppe with the groups of the Caucasus Paleolithic and Mesolithic populations much earlier.^31^^,^^32^ If this was the case, the individuals buried at the Nalchik cemetery and the Darkveti-Meshoko population represent at least the second wave of the southern population into the Western and Northern Caucasus and the steppe in the first half of the fifth millennium BC. The closest analogies to Darkveti_Meshoko ceramics come from sites of the Sioni type in the South Caucasus (first half of the 5th millennium BCE—beginning of the 4th millennium BCE),^33^ but they lack stone bracelets—the symbolic type for all Darkveti-Meshoko sites. It can be assumed that in addition to the route through the area of sites such as Sioni, there was another, still unknown to us, route of cultural influence on the Western and Northern Caucasus, passing through the coastal regions of the Black Sea (Trebizond–Adjara). For the Chalcolithic period, these areas still remain an archaeological blank spot.^34^

In the end of the fifth millennium BC—beginning of the fourth millennium BC, this wave was followed by another one (Chaff-face ware complex)^35^ which is linked finally to the emergence of the Maykop culture in the Northern Caucasus. From the archaeological point of view, the latest Darkveti-Meshoko sites are linked to the Maykop culture in the Northern Caucasus and the late sites of the Sioni type in the Southern Caucasus^36^; however, it is not yet possible to say to what extent the Trans-Caucasus routes of the fifth and the fourth millennia BCE overlap.

Finally, we cannot rule out that at the beginning of the third millennium BC when the Maykop culture ceased to exist, in the Western Caucasus both the Maykop culture (Novosvobodnaya variant) and Darkveti-Meshoko sites were succeeded by the Dolmen culture, while in the Northern Caucasus, it was succeeded by a new population, probably, of the southern origin, i.e., the Northern Caucasus culture. It is the influence of the latter culture on the Yamnaya population in the adjacent steppe rather than the influence of the Maykop culture that manifests itself through archaeological finds more vividly.

We can infer from this analysis that the history of appearance of the population that had components of Caucasus and West Asian ancestry in the Northern Caucasus and the steppe is much more complex than is now believed.^9^^,^^37^ Given this context, the genetic profile of the human from the Nalchik cemetery is an important element of the puzzle that has to be solved to get a complete paleogenetic picture in the Caucasus and the adjacent regions north and south of the Caucasus at the beginning of the fifth millennium BCE.

### Limitations of the study

This study has potential limitations in terms of empirical evidence, as there are only a limited number of relevant archaeological samples available for genetic analysis. The Nalchik cemetery initially had 121 burials. However, only two of these burials were preserved and included in the museum collection as exhibition samples. These two burials are known as burial 86 and burial 42, the latter being significant due to a craniotomy. The State Hermitage Museum provided the sample from burial 42 for paleogenetic analysis.

Despite these limitations, we made use of all the available data, which collectively support the hypothesis we put forward.

To conduct more robust and impactful research, it is necessary to conduct additional paleogenetic analyses on samples from Khvalynsk, Darkveti-Meshoko, and Sioni type burial sites.

It is important to highlight that late Chalcolithic burials are extremely rare in the North and South Caucasus. Only with the discovery of new burials from this period will it be possible to conduct additional genetic analyses.

## Resource availability

### Lead contact

Further information and requests should be directed to the main contact, Prokhortchouk E.B. (prokhortchouk@gmail.com).

### Materials availability

This study did not generate new unique reagents.

### Data and code availability

Sequencing data have been deposited in the NCBI: PRJNA1074255. This paper does not report the original code. Any additional information required to reanalyze the data reported in this paper is available from the lead contact upon request.

## Acknowledgments

We thank the State Hermitage Museum and personally Yuri Piotrovskij for providing the sample for the paleogenetic analysis.

Funding statement.

This work was financially supported by state assignment of Federal Research Centre «Fundamentals of Biotechnology» of the Russian Academy of Sciences, Moscow, Russian Federation (Project Reg. No. 122041100149-7 and 124060400040-3).

## Author contributions

Conceptualization, E.P. and V.T.; K.Z. and M.L. performed the laboratory works; V.T. provided archaeological materials and associated information; F.S. analyzed data; E.P., V.T., K.Z., and F.S. writing – original draft; writing – review and editing, E.P., V.T., and F.S..

## Declaration of interests

The authors declare no competing interests.

## STAR★Methods

### Key resources table

**Table:** 

REAGENT or RESOURCE	SOURCE	IDENTIFIER
**Biological samples**		

Ancient human remains	This paper	NL1.2.2

**Chemicals, peptides, and recombinant proteins**		

5 M guanidine hydrochloride	Suzhou Yacoo Science	SYS-Y0010–0.5
Isopropanol	Biolabmix	MRP100
Sodium acetate	Sisco Research	SRL-74537-500G
Tween 20	Servicebio	SB-GC204002–1.0
uracil-DNA glycosylase	qiagen	G5010L
endonuclease VIII	qiagen	Y9080L
Silica magnetic beads	G-Biosciences	786–915
Isopropanol	Biolabmix	MRP100
Sodium acetate	Sisco Research	SRL-74537-500G
Tween 20	Servicebio	SB-GC204002–1.0
uracil-DNA glycosylase	qiagen	G5010L
endonuclease VIII	qiagen	Y9080L
Silica magnetic beads	G-Biosciences	786–915

**Critical commercial assays**		

ACCEL-NGS 1S Plus DNA Library Kit	Swift Biosciences	10096
KAPA HiFi HotStart Uracil+ReadyMix Kit	07959052001	HiFi
The MyBaits Expert Human Affinities Prime Plus Kit	Daicel Arbor Biosciences	DBA-352096
HiSeq 1500	Illumina	FC-401-4003
KAPA HiFi HotStart Uracil+ReadyMix Kit	07959052001	HiFi
The MyBaits Expert Human Affinities Prime Plus Kit	Daicel Arbor Biosciences	DBA-352096
HiSeq 1500	Illumina	FC-401-4003

**Deposited data**		

Sequencing Data	SRA	PRJNA1074255
Genotype Data	Reich Lab website	https://reich.hms.harvard.edu/datasets
Genotype Data	Reich Lab website	https://reich.hms.harvard.edu/datasets

**Software and algorithms**		

BBDuk	Bushnell B. et al., 2017^38^	https://jgi.doe.gov/data-and-tools/software-tools/bbtools/bb-tools-user-guide/bbduk-guide/
PALEOMIX version 1.2.14	Schubert et al., 2014^39^	https://paleomix.readthedocs.io/en/stable/
BWA v0.7.17	Li and Durbin, 2009^40^	http://bio-bwa.sourceforge.net/
samtools version 1.9	Li et al., 2009^41^	http://samtools.sourceforge.net/
pileupCaller	https://github.com/stschiff/sequenceTools	https://github.com/stschiff/sequenceTools
mapDamage v2.0	Jónsson et al., 2013^42^	https://ginolhac.github.io/mapDamage/
PLINK v1.90	Purcell et al., 2007^43^	http://zzz.bwh.harvard.edu/plink/
ADMIXTURE	Alexander et al., 2009^18^	http://dalexander.github.io/admixture/download.html
EIGENSOFT	Patterson et al., 2006^44^	https://github.com/DReichLab/EIG
GLIMPSE v1.0.0	Rubinacci et al., 2021^45^	https://odelaneau.github.io/GLIMPSE/glimpse1/index.html
ancIBD	Ringbauer et al., 2024^46^	https://github.com/hringbauer/ancIBD
PALEOMIX version 1.2.14	Schubert et al., 2014^39^	https://paleomix.readthedocs.io/en/stable/
BWA v0.7.17	Li and Durbin, 2009^40^	http://bio-bwa.sourceforge.net/
samtools version 1.9	Li et al., 2009	http://samtools.sourceforge.net/
pileupCaller	https://github.com/stschiff/sequenceTools	https://github.com/stschiff/sequenceTools
mapDamage v2.0	Jónsson et al., 2013^42^	https://ginolhac.github.io/mapDamage/
PLINK v1.90	Purcell et al., 2007^43^	http://zzz.bwh.harvard.edu/plink/
ADMIXTURE	Alexander et al., 2009^18^	http://dalexander.github.io/admixture/download.html
EIGENSOFT	Patterson et al., 2006	https://github.com/DReichLab/EIG
GLIMPSE v1.0.0	Rubinacci et al., 2021^45^	https://odelaneau.github.io/GLIMPSE/glimpse1/index.html
ancIBD	Ringbauer et al., 2024^46^	https://github.com/hringbauer/ancIBD

**Other**		

Allen ancient DNA resource (aadr) for previously published ancient and modern DNA data		https://dataverse.harvard.edu/dataverse/reich_lab

### Experimental model and subject details

#### Archaeological context

##### Darkveti-Meshoko Eneolithic culture and the Nalchik cemetery

The sites attributed to this culture occupy a large part of the Western Caucasus area which extends from Novorossiysk to the region of Kavkazskiye Mineralnye Vody (Caucasus Spar region) north of the Main Caucasian Range as well as the mountain and Black Sea coastal areas as far as the Likhi Range, including a part of Abkhazia, Mingrelia and Imereti. Darkveti in the southeastern part of the area (Western Georgia) and Meshoko in the northwestern part of the area (Kuban river basin) are the most prominent sites of this archaeological tradition.

##### History of discovery and investigations

In the mid-1960s, taking into account characteristics of pottery and widespread use of stone bracelets, A.A. Formozov came to the conclusion on cultural unity of the Kuban sites of the Meshoko type and the Western Georgian sites of the Tetramitsa and Sagvarjile type. He then attributed the Nalchik cemetery to the same cultural contex.^10^^,^^47^ Subsequently, examining the multilayer Darkveti rock shelter in Western Georgia, A.F. Nebieridze not only confirmed A.A. Formozov’s observations but also suggested that the Kuban sites of the Meshoko type, the Western Georgian sites of the Darkveti type and the Black Sea coastal sites of the Myskhako and Ochazhny Grot types (Vorontsovskaya Cave) should be referred to the same Western Caucasus Eneolithic culture that had local variants.^48^

Initially, the sites of the Meshoko type were erroneously identified with the Maykop culture which was known only by reference to burials in kurgans^49^; however, by the early 1990s it was found that those sites dated to an earlier period and were associated with a separate archaeological tradition.^25^^,^^50^^,^^51^^,^^52^

##### Types of sites

Presently, around 30 Darkveti-Meshoko sites are known. In most cases, these are unfortified and fortified settlements of various types, camps in rock shelters, caves and overhangs. Typically, they have wattle and daub constructions. Some settlements are fortified.^49^^,^^51^ The Nalchik site is the only known cemetery. Three more burials have been found in the occupation layers at settlements (Skala, Unakozovskaya Cave, and Sagvarjile).

##### Economy

Judging by the finds of grain and bones of domesticated animals, the economy of the sedentary population was based on agriculture (*Triticum aestivum, Hordeum vulgare* subsp. *Distichum Korn*), raising of pigs and cattle.^53^^,^^54^^,^^55^

##### Tools and Implements

With some differences revealed, the comparative analysis of the materials from the settlements show a high degree of typological similarity, in particular, because of uniformity of form, quality and ornamentation of ceramics (round-bottomed burnished vessels made of clay tempered with minerals prevail), extensive use of serpentine, slate and ceramic bracelets, a special type of ornamented spindle whorls, flint arrowheads and segments, stone polished chisel axes and adzes of smaller size as well anthropomorphic and zoomorphic sculptures.^13^^,^^22^^,^^25^^,^^51^^,^^53^^,^^56^^,^^57^^,^^58^^,^^59^^,^^60^

##### Periodization

The issue related to the identification of the Darkveti-Meshoko development stages has not yet been adequately addressed; however, some sites have revealed a set of clear typological characteristics that differentiate one stratigraphic level from another.^61^^,^^62^ According to the stratigraphy and co-occurrence of ceramics types, changes over time in the number of microliths, the Darkveti-Meshoko culture had at least two stages in its development. At an early stage the sites of this culture, with the exception of the mountain and coastal areas, were located in the plain areas in the Northern Caucasus piedmont. The sites preceding the Darkveti-Meshoko culture are not known in the Northern Caucasus. According to A.F. Nebieridze, in Western Georgia they are preceded by Neolithic sites of the Anaseuli II type^63^; however, geographically they are situated in different landscape zones. North of the Caucasus the early sites of the Svobodnoye–Zamok–Darkveti–Dzudzuana type chronologically date to the same period as the Khvalynsk and Early Sredny Stog sites as well as the sites of the Novodanilovka type and the Tripolye sites of the B I period.

During the second development stage the area occupied by the culture was reduced because of the spread of the Maykop sites in the Northern Caucasus piedmont. High quality ceramic vessels found at 10 Darkveti-Meshoko settlements demonstrate that during that stage both cultures coexisted. In the Eastern European steppe the cultures contemporary with the Late Tripolye sites (Tripolye СI – II) and the Late Sredny Stog cultures date to the same period. In the Southern Caucasus ceramics from the sites of the Sioni type demonstrate partial similarity with the Darkveti-Meshoko ceramics.

The Darkveti-Meshoko culture ceased to exist when the Dolmen culture and the sites of the Ochamchire settlement type appeared in the Western Caucacus.

##### Chronology

Given the relative chronological position of the Darkveti-Meshoko sites in the system of the Caucasus prehistoric antiquities as well as results of their radiocarbon dating, on the whole, the culture can be approximately dated to the period c. 5000/4900–3300/3200 BC.^64^^,^^65^

##### Local variants

While the similarities clearly prevail over the differences, the latter can be used as a criterion for singling out at least two local variants, i.e., Meshoko and Darkveti, distinctive traits of which are mainly due to specific characteristics of external links of this archaeological culture in the west and in the east of the area it occupied.

##### Origin

The origin of the Darkveti-Meshoko culture is still unclear. The closest analogies to Darkveti_Meshoko ceramics come from sites of the Sioni type in the South Caucasus (first half of the 5th millennium BC - beginning of the 4th millennium BC),^26^ but they lack stone bracelets - the symbolic type for all Darkveti-Meshoko sites. It can be assumed that in addition to the route through the area of sites such as Sioni, there was another, still unknown to us, route of cultural influence on the Western Caucasus, passing through the coastal regions of the Black Sea (Trebizond - Adjara). For the Chalcolithic period, these areas still remain an archaeological blank spot.^34^

Judging by the forms, quality and elements of ceramic ornamentation it seems that the ceramic tradition of Darkveti - Meshoko and Sioni goes back to the tradition characteristic of ceramics from North Mesopotamian sites dating to the final period of the Early Chalcolithic–beginning of the Late Chalcolithic.

##### Nalchik cemetery (43.482 N, 43.607 E)

The cemetery without mounds included 121 burials (Figures S1 and S2), it was located within the limits of the city of Nalchik (Republic of Kabardino-Balkaria, Russia) and was excavated in 1929. The archaeological collection is kept in the Kabardino-Balkaria National Museum (Nalchik) (finds) and the State Hermitage Museum (finds and remains of two skeletons from the graves 42 and 86); the field documentation is kept in the scientific archives of the Institute for the History of Material Culture, RAS (Saint-Petersburg). The results of the excavations were published in 1941.^66^ The radiocarbon dates of the skeleton from burial 86 (Figure S3), put the occupation of this site around the early fifth millennium BC (Table S1).

The State Hermitage Museum provided one sample for the paleogenetic analysis (burial no. 42), it is a human tooth, left upper molar M3 (Collection of the State Hermitage Museum, No. 447/D-15).

The skeleton of the buried individual (a male of 40–60 years) with legs flexed, on his the right side, with the arms extended along the body. The skull was trephined. The bones of the legs were destroyed by a latest trench running across the cemetery. The entire skeleton was sprinkled with ocher, in particular, the skull and the shoulder and elbow bones. The skeleton was overlaid by burial 41, and in its turn, it overlaid burials 44 and 45.

### Method details

#### DNA preservation

To isolate aDNA, tooth sample of an adult individual (identification number NL) were collected. Tooth powder weights were obtained from the samples with the corresponding identification numbers NL1.1, NL1.2, and NL1.2.2, from which DNA was isolated and libraries of single-stranded fragments were prepared for the initial shotgun sequencing in order to assess endogeneity. Sequencing results are presented in Table S3.

The NL1.2.2 sample was characterized by the highest proportion of the frequency of cytosine to thymine substitutions at the 5′ ends of DNA fragments and was selected for further in-solution enrichment and sequencing. The frequency of C to T substitutions around the 5′ ends of the sequences are presented in the Figure S4.

#### DNA isolation and genomic library preparation

All experiments with aDNA were carried out in “a clean room” – a room specially equipped for these purposes at the Federal Research Center “Fundamentals of Biotechnology” of the Russian Academy of Sciences (Skryabin Institute of Bioengineering).

To isolate aDNA from the samples provided for genetic analysis, we obtained three portions of tooth powder weighing 50, 78 and 110 mg. DNA was isolated by magnetic separation using buffer D that of the Dabney method (5 M guanidine hydrochloride, 40% (v/v), isopropanol, 0.12 M sodium acetate, and 0.05% (v/v) Tween 20) and silica-coated magnetic beads.^67^

The resulting DNA was used to prepare libraries of single-stranded DNA fragments using the ACCEL-NGS 1S Plus DNA Library Kit (Swift Biosciences, USA) according to the original protocol but with minor modifications: for the steps providing strand elongation and sample indexing, uracil-tolerant polymerase (KAPA HiFi HS Uracil+RM, USA) was used. To assess the content of endogenous DNA, test sequencing of the constructed libraries of low-coverage DNA fragments was carried out, approximately 3–4 million single reads per sample (50 bp long). For the sample with the best preservation of the genetic material (high endogeneity and the presence of C > T substitutions at the 5′ ends of DNA fragments), an additional library was prepared from the same DNA extract and pre-treated with a mixture of uracil-DNA glycosylase (UDG) and endonuclease VIII.^68^ The mixture of enzymes made it possible to remove uracil from the aDNA strands and turn the resulting abasic sites into single nucleotide breaks, while some of the uracils at the ends of the fragments were preserved, which is associated with the low efficiency of enzymes in these regions. The removal of uracils improved the quality of mapping and prevented a distortion of the results of the subsequent statistical processing.^69^

The MyBaits Expert Human Affinities Prime Plus Kit (Daicel Arbor Biosciences) was used for subsequent enrichment for the genome regions of interest. Biotinylated single-stranded DNA probes from the kit cover single nucleotide polymorphisms (SNPs) from the panel “1240K capture”,^19^ 46 thousand additional unique SNPs of the Y chromosome of known haplogroups according to the classifier of the International Society of Genetic Genealogy (ISOGG),^70^ and a set of MitoTrio probes for three different mitochondrial genomes: the Revised Cambridge Reference Sequence (rCRS), the Reconstructed Sapiens Reference Sequence (RSRS), and the Vindija Neanderthal sequence (GenBank NC_011137).^71^ Libraries were sequenced on a HiSeq 1500 instrument (Illumina, USA) in paired read mode 2 × 150 bp for genome-wide sequencing and in the mode of single readings 50 bp long for test libraries.

### Quantification and statistical analyses

#### Bioinformatics analysis

To remove contaminating DNA reads from the sequencing data, we used the BBDuk software^38^ included in the BBMap package, and bacteria, fungi, plants, viruses, and other organism databases. The output of the BBDuk tool was analyzed using the PALEOMIX pipe-line (version 1.2.14).^39^ Sequencing adapters were trimmed using the Cutadapt v3.4 tool.^72^ Sequences were aligned to the reference human genome sequence (hg19/GRCh37) using BWA (version 0.7.17).^40^

Aligned reads were filtered to ensure a minimum display quality of 20 using samtools view (version 1.9).^73^ Indexing, sorting, and removal of duplicates (rmdup) were performed using the samtools tool (version 1.9).^73^ To call genotypes from aligned reads, a PileupCaller (https://github.com/stschiff/sequenceTools) with the “–randomHaploid” mode was used, which calls haploid genotypes by randomly selecting one high-quality base (phred base quality score ≥30) on the 1240K SNP panel (https://reich.hms.harvard.edu/).

Postmortem DNA damage patterns were analyzed using the MapDamage2 software,^42^ which offers a series of tools for imaging and modeling postmortem damage patterns observed in ancient samples. MapDamage2.0 also makes it possible to recalculate base quality scores in order to mitigate the impact of postmortem damage on further analysis.

To determine the genetic clustering of the NL1.2.2 sample among the ancient samples lf? known at the time of the study presented in the Allen Ancient DNA Resource (AADR) panel,^74^ the ADMIXTURE v.1.3.0 software^18^ was used. SNPs were trimmed for sites with linkage disequilibrium using PLINK v1.9.^43^ The sliding window was 50 SNPs; the step was 5 SNPs; the r2 threshold was 0.2 (–in-dep-pairwise 50 5 0.2). There were 10 runs with random starting values for a number of clusters (*K*) in the range of 4–12; the run with the lowest cross-validation error was selected to plot the graph of population admixture.

For principal component analysis (PCA), the smartpca tool from the EIGENSOFT package was used. Ancient samples were projected onto the first two components of the modern samples. A list of samples is presented in Table S2. The following parameters were set by default: lsqproject: YES, numoutlieriter; 0, shrinkmode; and YES for the smartpca analysis. Mitochondrial haplotypes were determined using the HaploGrep program.^75^ Determination of Y chromosome haplogroups was carried out by comparing alleles on the phylogenetic tree ISOGG version 15.73. F4-statistics were calculated using the qpDstat program from the ADMIXTOOLS software package with default parameters. All constructions were based on available data obtained from whole genome sequencing of the samples. To model the genome from the components of ancestral populations, we used the qpAdmix programs with the “allsnps: YES” parameter and "Papuan.DG", "Han.DG", "Chukchi.DG", "Russia_Ust_Ishim_HG_published.DG", "Kostenki14", "ONG.SG", "Yoruba.SDG", "Mbuti.SDG", "Karitiana.SDG" and "Iran_Ganj_Dareh_Neolithic" were chosen as the right populations.

We utilized the imputation and phasing tool GLIMPSE v.1.0.0^45^ and the 1000 Genomes phase 3^76^ as a reference panel to impute the ancient genomes. First, we used GLIMPSE_chunk to split chromosomes into chunks of sizes in the range 1–2 Mb and included a 200-kb buffer region at each side of a chunk. These chunks were then imputed using GLIMPSE_phase, with the parameters --burn 10, --main 15, and --pbwt-depth 2. And then, we ligated the imputed chunks with GLIMPSE_ligate. We used ancIBD^46^ to detect genomic segments common to the selected ancient individuals in this study.

## Supplemental information

Document S1. Figures S1–S5 and Tables S1, S3–S6

Table S2. A list of samples for PCA and Admixture analyses, related to Figure 2–3

Table S7. F4-statistics in the form of (Yoruba, West Eurasian steppes; Unakozovskaya, Nalchik), related to STAR Methods

Table S8. Outgroup F3 statistics Related to STAR Methods and Figure S5

Table S9. Modeling of the Nalchik Man genome, related to Figure 5A Individuals from the Unakozovskaya cave and Armenian Chalcolithic representatives

Table S10. Modeling of the Nalchik Man genome, related to STAR Methods

Table S11. Modeling the ancestry of individuals from Khvalynsk II, related to Figure 5B

Table S12. Modeling of the Nalchik Man genome, related to Figure 5B Individuals from the Unakozovskaya cave and Armenian Chalcolithic representatives

Table S13. Modeling the ancestry of individuals from Khvalynsk II, related to Figure 5B

Table S14. IBD analysis of Khvalynsk, Unakozovo and Nakchik samples, related to Figure 6
