# Supplementary material for: Human DNA from the oldest Eneolithic cemetery in Nalchik points the spread of farming from the Caucasus to the Eastern European steppes
Source: iScience. 2024 Oct 16;27(11):110963. doi: 10.1016/j.isci.2024.110963 (PMC11576401; doi:10.1016/j.isci.2024.110963)
Supplement: Document S1. Figures S1–S5 and Tables S1, S3–S6 [file mmc1.pdf]

iScience, Volume *27*

Supplemental information

Human DNA from the oldest Eneolithic cemetery in Nalchik points the spread of farming from the Caucasus to the Eastern European steppes

K.V. Zhur, F.S. Sharko, M.V. Leonova, A. Mey, E.B. Prokhortchouk, and V.A. Trifonov

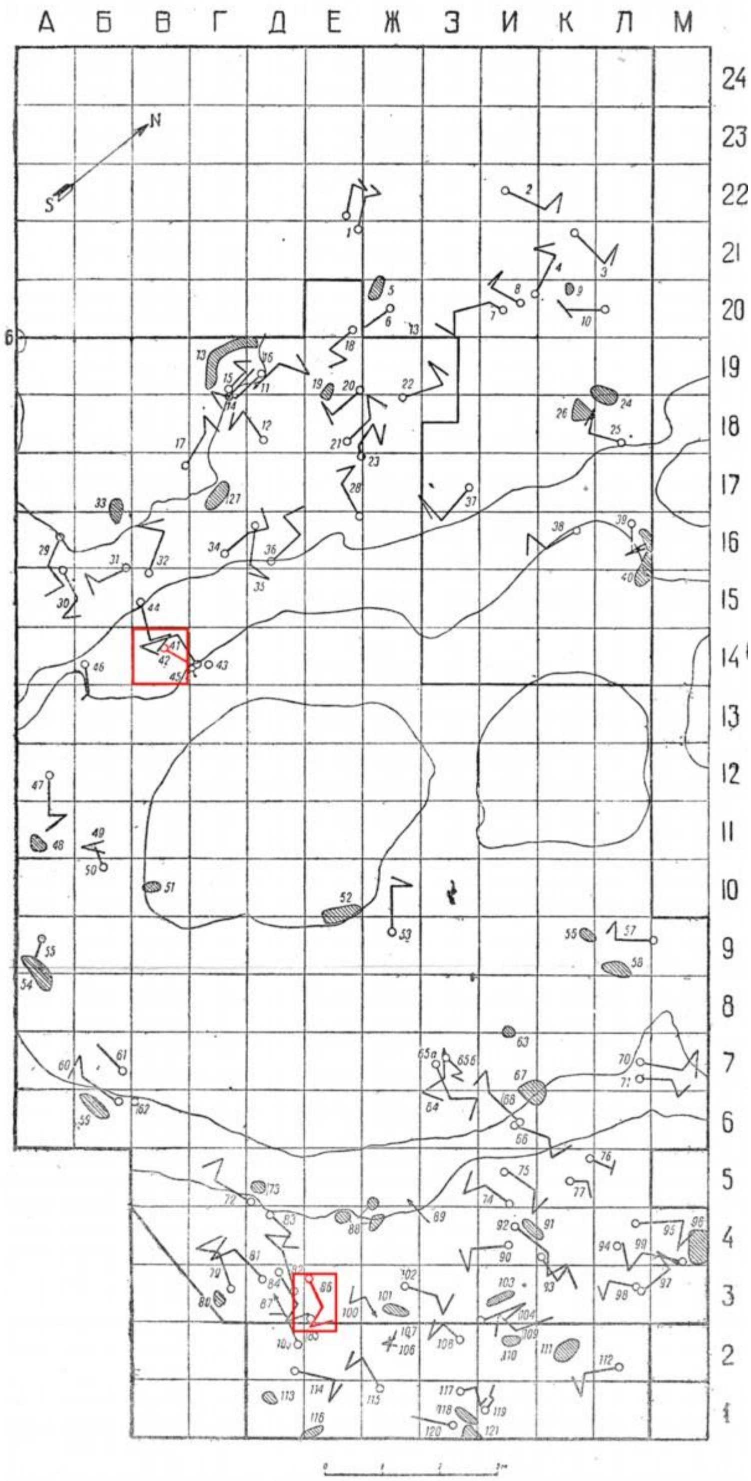

Figure S1. The graveyard in Nalchik. Related to STAR Methods.
General layout, the graves 42 (gene sequenced), 86 (AMS-dated) (red filling).

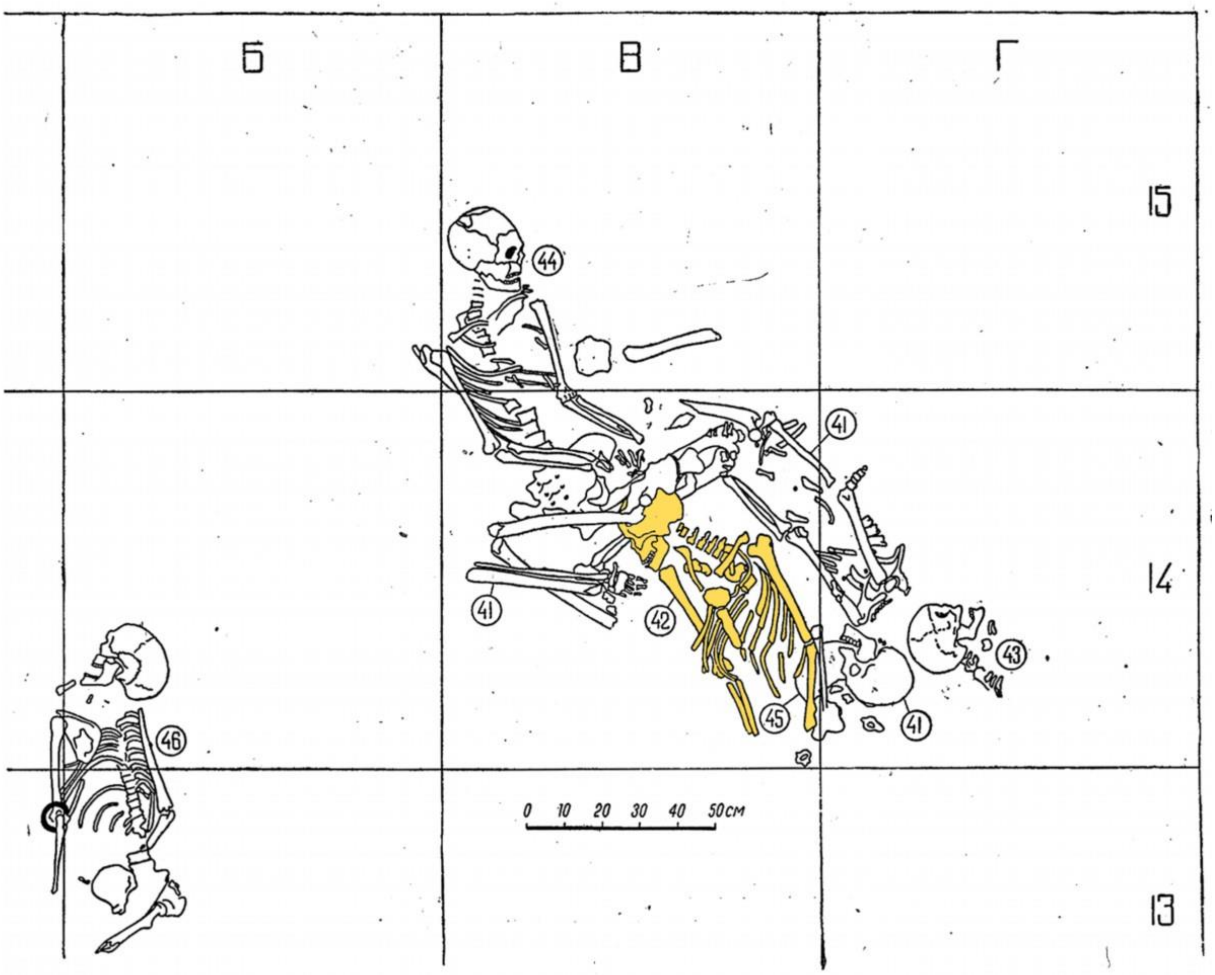

Figure S2. The graveyard in Nalchik. Related to STAR Methods. The grave 42 (yellow filling).

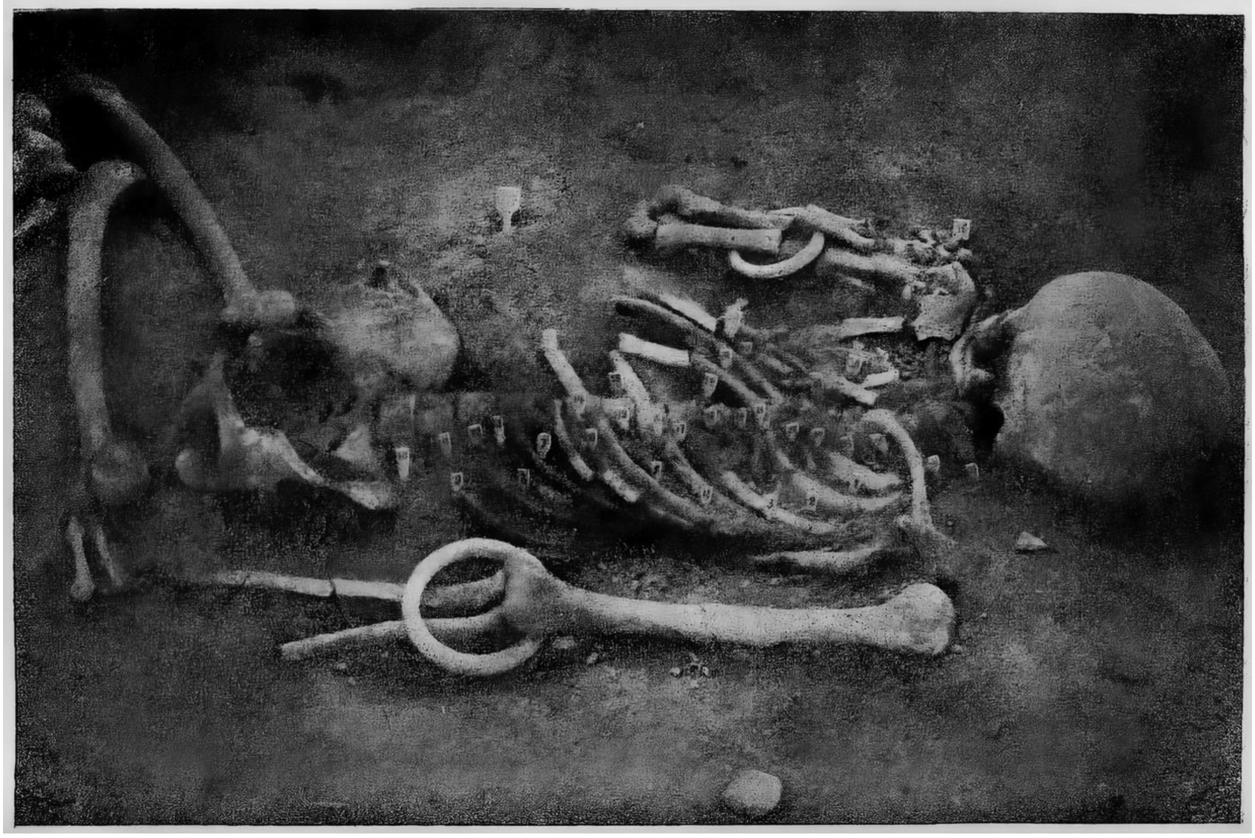

Figure S3. **The graveyard in Nalchik**. **Related to STAR Methods.** The grave 86.

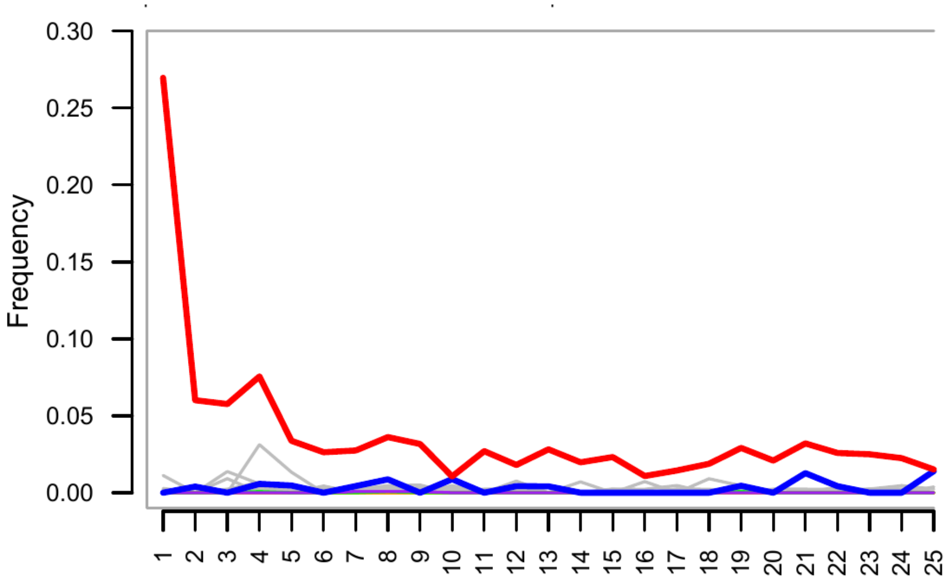

Figure S4. Post-mortem modifications at the 5' ends. Related to STAR Methods.
Frequency of C to T substitutions at the 5' ends of aDNA fragments for the NL1.2.2 sample.

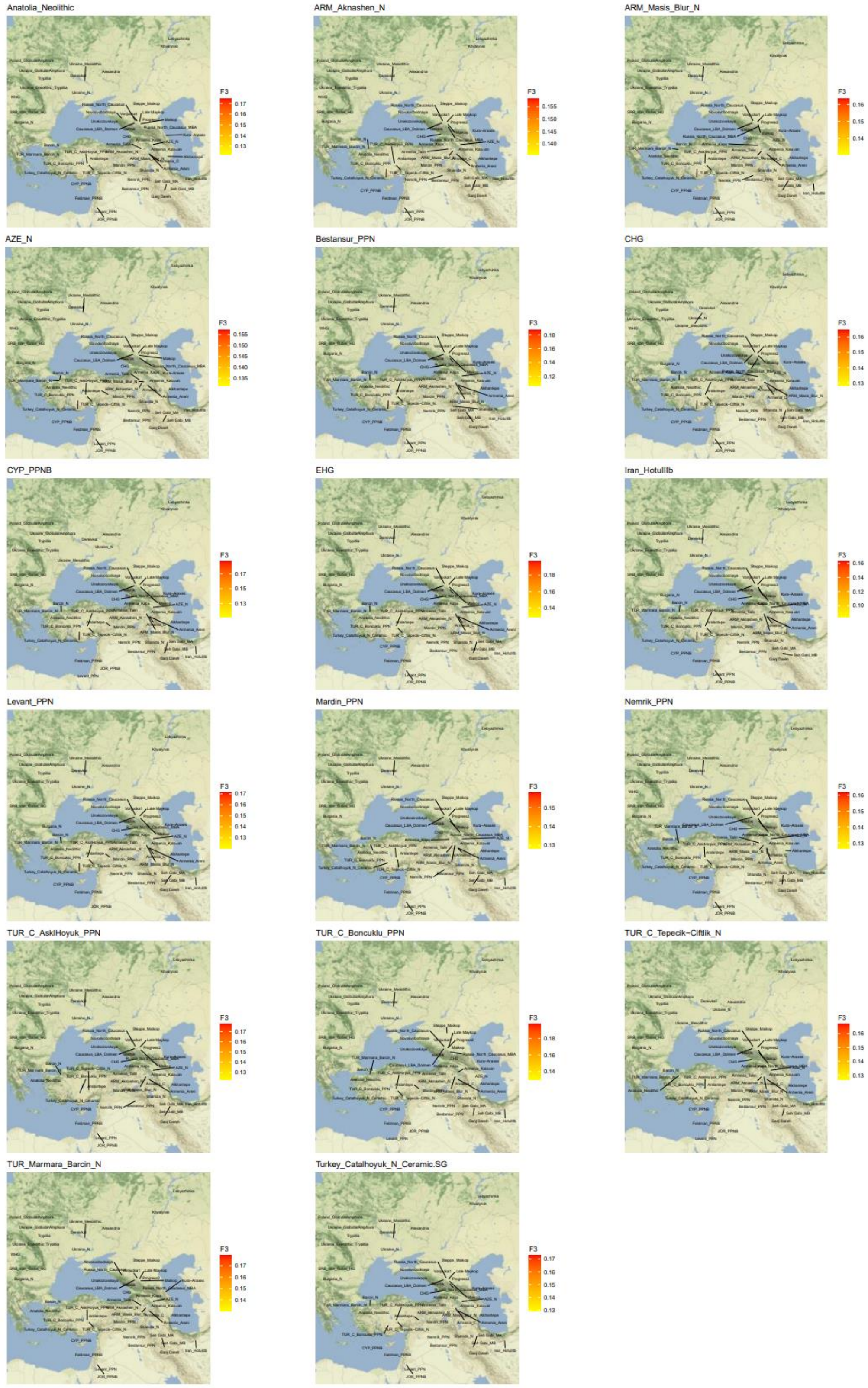

Figure S5. The geographical distribution of outgroup F3 statistics to measure the genetic closeness of populations to Darkweti-Meshoko and other cultures (test). Related to STAR Methods and Figure 4. Outgroup F3 statistics (Test, Candidates; Yoruba) were obtained using Candidate genomes from Steppe (EHG), Caucasus (CHG and Iran_HotuIIIb) and Neolithic populations. Higher F3 statistics (red colours) indicate more shared drift with the respective group in Candidates. All F3 statistics can be found in Supplementary Table 13.

Table S1. The radiocarbon dates. Related to STAR Methods. The occupation of this site around the early fifth millennium BC.

Lab Code	RCY BP	(CalBC) 68.2% (OxCal 4.2.3)
GrA-24442	5910±45	4836-4721
OxA-5561	6065±60	5197-4850

Table S3. Results of test sequencing of aDNA fragment libraries. Related to STAR Methods. Sequencing of test statistics.

ID of the library	Original number of reads	Number of reads after filtering	Number of reads mapped on hg19	After removal of PCR duplicates	% of endogenous DNA
NL1.1	670504	514475	21719	14624	2.84
NL1.2	508040	445456	6941	4832	1.08
NL1.2.2	870608	716725	12746	8817	1.23

Table S4. Results of sequencing. Related to STAR Methods. Result of genome-wide sequencing of the library of NL1.2.2

ID of the library	Original number of reads	Number of reads after filtering	Number of reads mapped on hg19	After removal of PCR duplicates	Coverage	% of endogenous DNA	SNP	sex	mt Haplogroup	Y Haplogroup
NL1.2.2	155602585	155492610	121349018	2390944	0,05	1,54%	452778	M	T2c1a1	R1b1

Table S5. Related to STAR Methods. Contamination results. Result of assessing the contamination of the sample according to such parameters as the degree of heterozygosity of X-chromosome with mathematical methods «MOM» (Methods of Moments) and «ML» .

ID of the library	nSNP	MoM	SE(MoM)	ML	SE(ML)
NL1.2.2	1457	18,64%	1,13E-02	19,00%	2,33E-14

Table S6. Heterozigotisty MT. Related to STAR Methods. Result of assessing the contamination of the sample according to the degree of mtDNA heterozygosity.

ID of the library	Heterozigotisty MT	X/Y
NL1.2.2	0,91%	1,793267617
